# Quantitative comparison of SARS-CoV-2 nucleic acid amplification test and antigen testing algorithms: a decision analysis simulation model

**DOI:** 10.1186/s12889-021-12489-8

**Published:** 2022-01-13

**Authors:** Phillip P. Salvatore, Melisa M. Shah, Laura Ford, Augustina Delaney, Christopher H. Hsu, Jacqueline E. Tate, Hannah L. Kirking

**Affiliations:** 1 COVID-19 Response Team, Centers for Disease Control and Prevention (CDC), 1600 Clifton Road NE, Atlanta, USA; 2 Epidemic Intelligence Service, Centers for Disease Control and Prevention (CDC), 1600 Clifton Road NE, Atlanta, USA

**Keywords:** SARS-CoV-2, COVID-19, Decision analysis, Antigen test, Mathematical model

## Abstract

**Background:**

Antigen tests for SARS-CoV-2 offer advantages over nucleic acid amplification tests (NAATs, such as RT-PCR), including lower cost and rapid return of results, but show reduced sensitivity. Public health organizations recommend different strategies for utilizing NAATs and antigen tests. We sought to create a framework for the quantitative comparison of these recommended strategies based on their expected performance.

**Methods:**

We utilized a decision analysis approach to simulate the expected outcomes of six testing algorithms analogous to strategies recommended by public health organizations. Each algorithm was simulated 50,000 times in a population of 100,000 persons seeking testing. Primary outcomes were number of missed cases, number of false-positive diagnoses, and total test volumes. Outcome medians and 95% uncertainty ranges (URs) were reported.

**Results:**

Algorithms that use NAATs to confirm all negative antigen results minimized missed cases but required high NAAT capacity: 92,200 (95% UR: 91,200-93,200) tests (in addition to 100,000 antigen tests) at 10% prevalence. Selective use of NAATs to confirm antigen results when discordant with symptom status (e.g., symptomatic persons with negative antigen results) resulted in the most efficient use of NAATs, with 25 NAATs (95% UR: 13-57) needed to detect one additional case compared to exclusive use of antigen tests.

**Conclusions:**

No single SARS-CoV-2 testing algorithm is likely to be optimal across settings with different levels of prevalence and for all programmatic priorities. This analysis provides a framework for selecting setting-specific strategies to achieve acceptable balances and trade-offs between programmatic priorities and resource constraints.

**Supplementary Information:**

The online version contains supplementary material available at 10.1186/s12889-021-12489-8.

## Background

The COVID-19 pandemic, caused by the SARS-CoV-2 virus, continues to cause significant morbidity, mortality, and economic hardship worldwide. Diagnostic testing is a cornerstone of COVID-19 response strategies in the U.S. and globally [1, 2]. Nucleic acid amplification tests (NAATs, such as real-time reverse transcription–polymerase chain reaction [RT-PCR]) and antigen tests are used to diagnose current infection with SARS-CoV-2 virus. NAATs are sensitive tests for SARS-CoV-2 infection and are often utilized as “gold-standard” assays for the diagnosis of COVID-19 [3]. However, programmatic implementation of NAATs may face challenges, such as long turnaround times, which hampers the ability of testing programs to be used to interrupt transmission [4]. Additionally, NAATs often carry substantial costs associated with reagents, equipment, personnel training and salaries, and quality control. Antigen tests offer several advantages over NAATs for SARS-CoV-2 testing programs, including lower costs, point-of-care administration, and rapid return of results. In particular, use of serial antigen testing may provide benefits over NAATs for controlling outbreaks in some settings, such as congregate living facilities [5]. To expand COVID-19 testing availability, the U.S. government distributed 150 million antigen tests in 2020 [6].

Despite the advantages of lower costs and faster turnaround time, antigen tests are generally less sensitive than NAATs for diagnosis of COVID-19, particularly for persons without COVID-19 symptoms [3]. In many cases, it is recommended to confirm the results of antigen tests with the use of more sensitive NAATs [5]. Several strategies for the use of antigen tests and NAATs have been recommended by public health organizations such as the U.S. Centers for Disease Control and Prevention (CDC) [5], the World Health Organization (WHO) [7], and the European Centre for Disease Prevention and Control (ECDC) [8]. Depending on program goals, different strategies may be optimal for maximizing case detection, minimizing lost productivity, or minimizing the use of NAAT testing. To date, there has been no quantitative comparison of the expected performance and testing efficiency of these different strategies at various levels of prevalence. In this analysis, we evaluated the diagnostic performance and testing volumes of SARS-CoV-2 antigen and NAAT programs under six diagnostic algorithms using a simulation-based decision analysis approach.

## Methods

### Population and Model Structure

We evaluated outcomes of a modeled population of 100,000 persons seeking community-based SARS-CoV-2 testing (rather than facility-based serial testing) in settings of 5%, 10%, 15%, and 20% prevalence of SARS-CoV-2 infection. (Numerical results summarized in the text focus on the 10% prevalence level for conciseness.) Prevalence levels can vary substantially over time and geographically [9] and these levels of prevalence were selected as representative of the range of percent positivity by RT-PCR in a majority of U.S. states in March 2021 [10]. Model input parameter estimates were derived from antigen test evaluations in the U.S. from September to December 2020 (Table 1). Because these primary data were collected within U.S. populations, this analysis represents expected outcomes in a U.S. setting.

**Table 1:** Sampling distributions from empiric studies for model input parameters

Parameter	Point Estimate ^a^	Range ^a^	References
Percent of Cases Reporting Symptoms^b^ at Time of Testing	67%	54-84%	12–15
Percent of Non-Cases Reporting Symptoms^b^ at Time of Testing	32%	18-53%	12, 13, 15
Antigen Test Sensitivity Among Symptomatic^b^ Cases	80%	64-94%	12, 13, 15–17
Antigen Test Sensitivity Among Asymptomatic Cases	55%	41-69%	12, 13, 15–18
Antigen Test Specificity Among Symptomatic^b^ Non-Cases	99.7%	98.9-100%	12, 13, 15–17
Antigen Test Specificity Among Asymptomatic Non-Cases	99%	98.0-100%	12, 13, 15–18
NAAT Sensitivity for viral RNA Detection (including previously infectious persons) ^c^	100%		
NAAT Specificity ^c^	100%		
Sensitivity of Repeat Antigen Test (After Initial Negative Antigen Result)	18%	10-29%	13
Specificity of Repeat Antigen Test (After Initial Negative Antigen Result)	100%	99.8-100%	13
Proportion of Asymptomatic Non-Cases Reporting Recent Close Contact Exposure at Time of Testing	27%	9-45%	12,13
Mean Time Elapsed Between Sampling and Return of NAAT Result (days)^c^	3	1-5	

Abbreviations: NAAT – Nucleic Acid Amplification Test

^a^ Parameter values sampled from a triangular distribution with the modal value defined by the point estimate and upper and lower bounds defined by the range

^b^ Symptom criteria varied across reports used to estimate parameter values but were generally defined as the presence of one or more COVID-19 symptom at the time of testing

^c^Model assumption

**Fig. 1:**
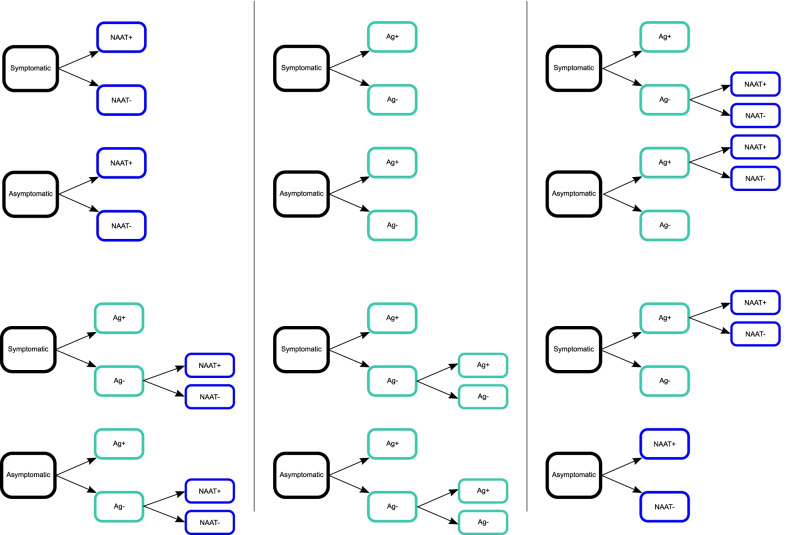
Modeled algorithms for SARS-CoV-2 NAAT and antigen testing. Each panel illustrates the testing strategy utilized for one of the modeled algorithms. Algorithm abbreviations and descriptions – **A*** NAAT Only*: each person tested receives a NAAT (such as an RT-PCR test); **B*** Ag Only*: each person tested a single antigen test; **C*** NAAT Confirmation for Sx/Ag-neg and Asx/Ag-pos*: each person receives an antigen test and NAAT is used to confirm diagnoses in persons for whom antigen results do not match binary symptom status (e.g., a symptomatic person whose antigen result is negative); **D*** NAAT Confirmation of Ag-*neg: each person receives an antigen test and NAAT is used to confirm negative antigen test results; *(E) Repeat Ag Confirmation of Ag-*neg: each person receives an antigen test and, for those with initial negative results, a repeat antigen test (performed within approximately 30 min of the initial test) is used to confirm negative diagnoses; **F*** NAAT for Asx & Sx/Ag-pos*: – asymptomatic persons receive a NAAT, while symptomatic persons receive an antigen test followed by a NAAT for those with positive antigen results

We evaluated six diagnostic algorithms which were adapted from current recommendations for SARS-CoV-2 antigen testing in various settings. These algorithms are illustrated in Fig. 1 and can be summarized as follows: *(A) NAAT Only* – each person is tested for SARS-CoV-2 infection by a NAAT. *(B) Antigen (Ag) Only* – each person is tested using a single antigen test, the result of which is used as a definitive diagnosis. This algorithm represents settings with access to point-of-care antigen tests, but no access to NAAT. *(C) NAAT Confirmation for Symptomatic Antigen-Negative (Sx/Ag-neg) and Asymptomatic Antigen-Positive (Asx/Ag-pos) Results* – each person receives an antigen test and NAAT is used to confirm diagnoses in persons for whom antigen results do not match binary symptom status (e.g., a symptomatic person whose antigen result is negative). *(D) NAAT Confirmation of Negative Antigen Results (Ag-neg)* – each person receives an antigen test and NAAT is used to confirm negative antigen test results. *(E) Repeat Antigen Confirmation of (Ag-neg)* – each person receives an antigen test and, for those with initial negative results, a repeat antigen test (performed within approximately 30 min of the initial test) is used to confirm negative diagnoses. *(F) NAAT for Asymptomatic Persons (Asx) & Symptomatic Persons with Positive Antigen Results (Sx/Ag-pos)* – asymptomatic persons receive a NAAT; symptomatic persons receive an antigen test followed by a NAAT for those with positive antigen results.

### Parameterization and Sampling

Parameters from empirical studies used for model simulations are summarized in Table 1. Antigen test sensitivity and specificity were assumed to be conditional on the binary symptom status of the person evaluated [11]; the prevalence of symptoms was modeled independently for infected and uninfected populations. We made the parsimonious assumption that sensitivity and specificity of NAATs are 100% as NAATs are typically considered the “gold standard” for diagnosis of SARS-CoV-2 infection. Sensitivity and specificity of repeat antigen testing were assumed to be conditional upon negative initial antigen results. Mathematical definitions for algorithms are detailed in the Supplementary Methods and Supplementary Table S1.

Parameters were sampled from triangular distributions (defined by a modal value and upper/lower bounds, characterized in Table 1) using Latin hypercube sampling to generate 50,000 simulations of each algorithm at each prevalence level. Outcomes are reported as the median and 95% uncertainty range (UR) of simulations for each scenario. URs can be interpreted as the range of outcomes that can be expected for algorithms under the most- and least-optimistic scenarios described by input parameter ranges. All calculations and analyses were performed using R software version 4.0.2 (R Core Team, Vienna, Austria). Code for the algorithm simulations can be found on the CDC collaborative software GitHub site (https://github.com/CDCgov/SARS-CoV-2-NAAT-and-Antigen-Testing-Algorithms).

### Primary Outcomes

Primary outcomes of interest were numbers of missed cases (persons with SARS-CoV-2 infection who receive a definitive diagnosis of “uninfected” by antigen testing with no recommendation for additional testing), false positive diagnoses (uninfected persons with a definitive diagnosis of “infected” by antigen testing with no recommendation for additional testing), and numbers of antigen tests and NAATs performed per 100,000 persons evaluated. Secondary outcomes (including person-time of lost productivity) and sensitivity analyses are available in the Supplementary Materials. Positive and negative predictive values of each algorithm are depicted in Supplementary Figure S1.

### Incremental Outcomes and Trade-Off Analysis

To characterize the potential consequences of adopting different testing algorithms in settings of varying NAAT capacity, we calculated [compared to the *(A) NAAT Only* algorithm] the incremental number of missed cases and saved NAATs [how many fewer NAATs were needed]under each algorithm. These incremental measures, calculated as a quotient representing the number of NAATs saved for each additional missed case compared to the *(A) NAAT Only* algorithm, provide an indication of the number of NAATs saved under different algorithms and the consequent trade-off of additional missed cases.

A similar incremental outcome was evaluated by comparing different testing algorithms to the *(B) Ag Only* algorithm and calculating the number of additional NAATs needed and consequent trade-off of additional cases detected. These measures are also presented as a quotient representing the number of additional NAATs needed for each additional case detected.

## Results

### Primary Outcomes

**Fig. 2:**
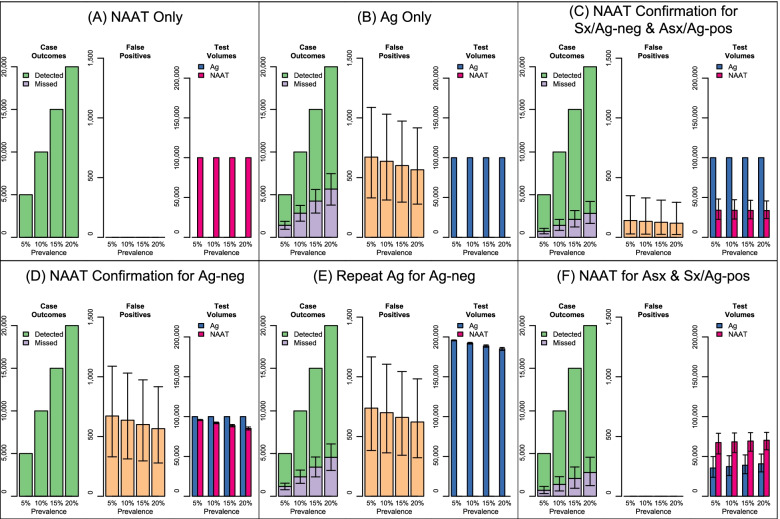
Primary outcomes (missed cases, false positives, and test volumes) of SARS-CoV-2 testing algorithms. Each panel presents the primary outcomes for one of the six algorithms investigated across four levels of prevalence. The left-hand graph of each panel shows the number of detected cases (in green) and missed cases (in purple). Each column of the left-hand graph sums to the total number of infected cases at each prevalence level. The middle graph of each panel shows the number of false positive diagnoses. The right-hand graph of each panel shows the number of NAATs (in magenta) and antigen tests (in blue) used. Bars represent median values and error bars represent 95% Uncertainty Ranges

Primary outcomes for each algorithm are presented in Fig. 2, for settings with SARS-CoV-2 prevalence ranging from 5% to 20% among 100,000 persons evaluated. (Detailed results are available in Supplementary Table S2.) Across prevalence levels, missed cases were greatest for algorithms that did not confirm negative antigen results with NAATs, *(B) Ag Only* and *(E) Repeat Ag for Ag-neg.* At 10% prevalence, these algorithms resulted in 2830 missed cases [*(B) Ag Only* 95% UR: 1890-3740] and 2280 missed cases [*(E) Repeat Ag for Ag-neg* 95% UR: 1507-3067], respectively. Algorithms in which NAATs were performed prior to all definitive negative diagnoses [*(A) NAAT Only *and* (D) NAAT Confirmation of Ag-neg*]*,* resulted in zero missed cases (due to assumed 100% sensitivity of NAATs). The remaining algorithms in which some but not all negative antigen results are confirmed by NAAT [*(C) NAAT Confirmation for Sx/Ag-pos & Asx/Ag-neg *and *(F) NAAT Confirmation for Asx & Sx/Ag-pos*], resulted in intermediate numbers of missed cases. At 10% prevalence, these algorithms result in 1409 missed cases (95% UR: 815-2100) and 1389 missed cases (95% UR: 622-2280), respectively.

False positive diagnoses were greatest in algorithms in which positive antigen results were not confirmed by NAATs—* (B) Ag Only, (D) NAAT Confirmation for Ag-neg, *and* (E) Repeat Ag for Ag-neg***.** The first two of these algorithms resulted in identical numbers of false positive diagnoses (median=635 [95% UR: 311-1031] false positive diagnoses at 10% prevalence) as both consider initial positive antigen results as definitive, while *(E) Repeat Ag for Ag-neg* resulted in higher numbers (median=699 [95% UR: 361-1105] false positive diagnoses at 10% prevalence) due to false positive diagnoses following the repeat antigen test. Algorithms where NAATs were performed prior to all definitive positive diagnoses [*(A) NAAT Only *and* (F) NAAT Confirmation for Asx & Sx/Ag-pos*], resulted in zero false positive diagnoses (assumed 100% specificity of NAATs). The remaining algorithm [*(C) NAAT Confirmation for Sx/Ag-pos & Asx/Ag-neg*], where some but not all positive antigen results are confirmed by NAAT, resulted in low numbers of false positive diagnoses (median=134 [95% UR: 27-330] at 10% prevalence).

Total testing volume remained constant for *(A) NAAT Only*and *(B) Ag Only* algorithms, at 100,000 NAAT or antigen tests, respectively. Antigen testing also remained constant at 100,000 tests for* (C) NAAT Confirmation for Sx/Ag-neg & Asx/Ag-pos*and *(D) NAAT Confirmation for Ag-neg* algorithms. Antigen testing volume was highest for the *(E) Repeat Ag for Ag-neg* algorithm and varied depending on the number of initial negative antigen results and total volume ranged from a median of 185,100 tests (95% UR: 183,200-187,000) at 20% prevalence to a median of 195,700 tests (95% UR: 195,100-196,300) at 5% prevalence. Among algorithms using antigen testing, antigen testing volume was lowest for *(F) NAAT Confirmation for Asx & Sx/Ag-pos* and varied depending on the prevalence of symptoms among persons evaluated, ranging from a median of 35,500 tests at 5% prevalence (95% UR: 23,800-49,700) to a median of 40,700 tests at 20% prevalence (95% UR: 30,500-52,900). Among algorithms using NAATs, NAAT testing volume was lowest for *(C) NAAT Confirmation for Sx/Ag-neg & Asx/Ag-pos*: at 10% prevalence, a median of 34,100 NAATs were used (95% UR: 22,500-48,100). NAAT testing volume was higher for the *(F) NAAT Confirmation for Asx & Sx/Ag-pos* and the *(D)NAAT Confirmation for Ag-neg*: at 10% prevalence, a median of 68,300 (95% UR: 54,900-79,400) NAATs and 92,200 (95% UR: 91,200-93,200) NAATs were used, respectively.

### Incremental Outcomes and Trade-Offs

**Fig. 3:**
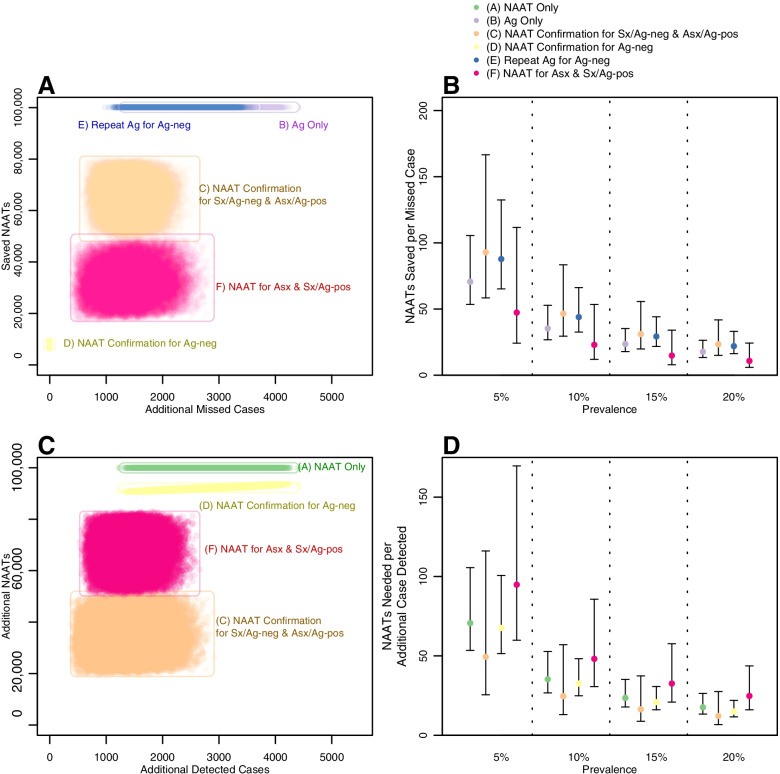
Trade-offs in algorithms for SARS-CoV-2 NAAT and antigen testing. Panel A depicts two primary outcomes (missed cases and NAAT volume) of 50,000 simulations for each of five algorithms compared to simulations of the (**A**)* NAAT Only* algorithm run under the same conditions at 10% prevalence in a population of 100,000 seeking testing. Panel **B** represents these results as a ratio of NAATs saved per missed case compared to the NAAT Only algorithm. The (**D**)* NAAT Confirmation for Ag-neg* algorithm results in zero missed cases, therefore this ratio equals positive infinity for all simulations and is not displayed. Panel **C** depicts missed cases and NAAT volume of 50,000 simulations compared to simulations of the (**B**)* Ag Only* algorithm run under the same conditions at 10% prevalence in a population of 100,000 seeking testing. Panel **D** represents these results as a ratio of NAATs needed per additional case detected compared to the Ag Only algorithm. In Panels **A** and **C**, each point represents the results of one simulation. In Panels **B** and **D**, points represent median values and error bars represent 95% Uncertainty Ranges. Algorithm (**E**)* Repeat Ag Confirmation of Ag-neg*, which utilizes no NAATs, is not displayed in Panels **C**-**D**

Incremental outcomes of simulations under algorithms compared to corresponding simulations under the *(A) NAAT Only*algorithm are depicted in Figure 3A (plotted as additional missed cases vs. NAATs saved, compared to *(A) NAAT Only*) at a level of 10% prevalence. The quotient of these measures is defined as the ratio of NAATs saved per additional missed case in Fig. 3B. The *(D) NAAT Confirmation for Ag-neg*algorithm had a ratio of positive infinity, resulting from zero additional missed cases (and a small number of NAATs saved). The *(C) NAAT Confirmation for Sx/Ag-neg & Asx/Ag-pos*algorithm had the most favorable ratio among remaining algorithms: at 10% prevalence, a median of 46 NAATs were saved per additional missed case (95% UR: 29-83) compared to *(A) NAAT Only*.

Incremental outcomes compared to the *(B) Ag Only*algorithm are depicted in Fig. 3C (plotted as additional cases detected vs. additional NAATs needed) at 10% prevalence. These measures are presented as a ratio of additional NAATs needed per additional cases detected in Fig. 3D. The *(E) Repeat Ag for Ag-neg* algorithm (which uses zero NAATs) had a ratio of zero additional NAATs needed per additional case. Among the remaining algorithms, the *(C) NAAT Confirmation for Sx/Ag-neg & Asx/Ag-pos*algorithm had the most favorable ratio: at 10% prevalence, a median of 25 NAATs were needed to detect each additional case (95% UR: 13-57) compared to *(B) Ag Only*. For both incremental outcomes, the order of algorithm favorability remained constant across prevalence levels; however, the absolute differences between algorithms shrank as prevalence increased. A summary and synthesis of algorithms to achieve a key programmatic priority, balancing missed cases and NAAT volume, is presented in Table 2; similar summaries for other programmatic priorities are presented in Supplementary Table S3.

**Table 2:** Summary and synthesis of algorithms for balancing missed cases and NAAT volume^a^

Algorithms^a^ to Consider	Pros^b^	Cons^b^	Synthesis	Impact of Prevalence
*(C) NAAT for Sx/Ag-neg & Asx/Ag-pos*	1. Moderate missed cases 2. Low NAAT volume 3. Low false positives	1. Moderate unneeded quarantine while waiting for results 2. High Ag volume	NAATs have the greatest accuracy but also greatest requirements in cost, time, personnel, and infrastructure. Programs often need to balance accuracy (case detection) and cost (NAAT volume). (D) results in no missed cases and saves between 4% NAAT test volume (at 5% prevalence) and 15% NAAT test volume (at 20% prevalence) relative to (A). (C) results in more missed cases but greatly reduces NAAT test volume (by 66%) compared to (A). This will save between 93 (at 5% prevalence) and 93 (at 20% prevalence) NAATs for each additional case missed. (E) eliminates NAAT entirely but substantially increases missed cases (23% compared to (A)). This will save between 87 (at 5% prevalence) and 22 (at 20% prevalence) NAATs for each additional case missed.	At low prevalence, cases are rare and many NAATs are needed for each case detected in (C), (D) and (E). As prevalence increases, cases increase more than NAAT volume increases and fewer NAATs are needed for each case detected in (C), (D) and (E). Absolute numbers of missed cases increase more and (E) than (C) (and remain 0 for (A) and (D)). As prevalence increases, the efficiency of (C), (D), and (E) becomes more favorable, while the negative consequences of (C) and (E) become less favorable.
*(D) NAAT for Ag-neg*	1. No missed cases	1. High false-positives 2. High NAAT volume 3. High Ag volume 4. High unneeded quarantine while waiting for results		
*(E) Repeat Ag for Ag-neg*	1. No NAAT infrastructure required 2. No unneeded quarantine while waiting for results	1. High missed cases 2. Highest false-positives 3. Highest Ag volume		
*(A) NAAT Only*	1. No missed cases 2. No false positives 3. No need for Ag testing infrastructure	1. Highest NAAT volume 2. Highest unneeded quarantine while waiting for results		

^a^ See Supplementary Table S3 for summary and synthesis of algorithms for other programmatic priorities

^b^See Methods and Fig. 1 for full descriptions of each algorithm evaluated. Algorithms are listed in order of favorability for balancing missed cases and NAAT volume. Algorithm (B), which does not implement NAATs, is excluded

§ Except where stated otherwise, numerical results are simplified by rank order for summary as follows: *Highest* refers to the algorithm for which the outcome is the highest number (compared to all other algorithms, across prevalence levels); *High* refers to algorithms which result in the second- or third-highest level outcome of algorithms evaluated; *Moderate* refers to the middle level of outcome (when outcomes from multiple algorithms are equal); *Low* refers to algorithms with result in the second- or third-lowest level of outcome; *Lowest* refers to the algorithm for which the outcome is lowest (when this lowest level is zero, this is stated as, e.g., “No missed cases”). See Results and Fig. 2 for exact numerical results

Abbreviations: NAAT – nucleic acid amplification tests (such as RT-PCR); Ag – antigen; Ag-pos - positive antigen result; Ag-neg - negative antigen result

## Discussion

In this analysis, we utilized a decision analysis approach to provide a quantitative comparison of different strategies for the use of antigen tests and NAATs in SARS-CoV-2 testing programs. The six algorithms evaluated reflect differing priorities testing in populations based on resources, SARS-CoV-2 prevalence, and tolerance for missed cases and false positives. Multiple reports have found that antigen tests are less sensitive than NAATs [12–18] and will result in some antigen false-negative results among cases. The *(A) NAAT Only* and *(D) NAAT Confirmation for Ag-*neg algorithms maximize the use of NAATs to confirm negative antigen results and yielded the smallest numbers of missed cases. However, use of confirmatory NAATs for negative antigen results also incurred a need for high NAAT capacity. A strategy that selectively confirms negative antigen results with NAAT was found to be the most efficient use of limited NAATs [*(C) NAAT Confirmation for Sx/Ag-neg & Asx/Ag-pos*]. When uninfected people are erroneously diagnosed with SARS-CoV-2 infection (due to false-positive results), consequent isolation orders and case investigations result in lost productivity, unnecessary use of limited public health resources, and, when resulting in co-isolation with true cases, puts them at risk for ongoing exposure. Therefore, algorithms which maximize NAATs to confirm positive antigen results yielded the smallest numbers of false-positive diagnoses [*(A) NAAT Only* and *(F) NAAT for Asx & Sx/Ag-pos*]. NAATs are often more costly to perform than antigen tests and may require logistical arrangements for timely off-site transport and testing. Strategies which minimize the use of NAATs [*(B) Ag Only* and *(E) Repeat Ag for Ag-neg*] offer benefits for resource-limited testing programs. Each of these algorithms may be advisable depending on the programmatic goals and resource limitations of community-based SARS-CoV-2 testing programs

Our analysis provides a quantitative framework for public health practitioners who are planning or evaluating community-based testing programs. A reference guide applying the results of our analyses to programmatic decisions, along with key priorities and indicators, is included in Table 2 and Supplementary Table S3. For programs intended to minimize missed cases, algorithms *(A) NAAT Only, (C) NAAT Confirmation for Sx/Ag-neg & Asx/Ag-pos*, and *(D) NAAT Confirmation for Ag-neg* are most preferable; selecting between these algorithms depends on tolerance for missed cases and available NAAT capacity. For programs intended to minimize NAAT volume, algorithms (*B) Ag Only*, *(C) NAAT Confirmation for Sx/Ag-neg & Asx/Ag-pos*, and *(E) Repeat Ag for Ag-neg* are most preferable; selecting between these algorithms depends on tolerance for missed cases and available NAAT and antigen test capacity. Predictive values (Supplementary Figure S1) can also provide key indicators of algorithm performance, particularly for individual and clinical decisions; however, programs should interpret predictive values with caution as algorithms with high predictive values may still result in unwanted outcomes (e.g., large numbers of missed cases) at the population level.

Each algorithm evaluated in this analysis is rooted in strategies currently recommended by public health organizations [except for (*A) NAAT Only*, an idealized baseline]. Each strategy recommended is articulated with important nuances; algorithms analyzed here are intended to be analogous to, but not exact reproductions of these strategies. Guidance from WHO and ECDC distinguishes strategies for antigen testing in communities with low and high prevalence of SARS-CoV-2 infection. In high prevalence settings, WHO recommends considering repeat antigen testing for those with negative results [7], analogous to *(E) Repeat Ag for Ag-neg*; ECDC indicates that negative tests should be confirmed with RT-PCR [8], analogous to *(D) NAAT Confirmation for Ag-*neg. In low prevalence settings following negative antigen results, WHO recommends clinical evaluation for suspect cases in lieu of confirmatory NAATs [7], analogous to *(B) Ag Only*; ECDC does not recommend antigen testing for asymptomatic persons and recommends confirmatory RT-PCR for symptomatic persons with positive antigen results [8], analogous to *(F) NAAT for Asx & Sx/Ag-pos*. CDC interim guidance recommends a unified strategy for testing across settings analogous to *(C) NAAT Confirmation for Sx/Ag-neg & Asx/Ag-pos* [5].

This decision analysis approach necessarily simplifies complex factors that may impact SARS-CoV-2 testing programs, and therefore results may not be representative of all testing programs. Our analysis does not account for individual-level variations (except symptom status) in test performance, such as patient age or sex. (However, empirical data indicate that these factors are not associated with significant differences in test performance [19]). This analysis is intended to be representative of community-based testing rather than facility-based serial testing (where each person is tested on a recurring basis). Our results would therefore overestimate the numbers of missed cases and testing volumes in serial testing programs. This analysis also does not evaluate dynamic transmission-related outcomes intrinsic to the intervention (as a consequence of detected/missed cases) which have been evaluated previously [20, 21]. Finally, this decision analysis approach is used to estimate expected outcomes under a theoretical perfect implementation of each algorithm to highlight the fundamental distinctions between testing algorithms (independent of implementation challenges). As a decision analysis model, our approach allows for a standardized comparison of the performance of all algorithms and, while specific settings or populations may differ from the one modeled, our conclusions about the relative benefits of each algorithm are portable for programmatic decisions across settings.

The results of our analysis are dependent on the accuracy and generalizability of the input parameter estimates used. Several reports have described the performance characteristics of several antigen tests, with comparable results across reports [12–18]. Programs implementing antigen tests with performance characteristics substantively different from the distributions described in Table 1 are likely to have different numbers of missed cases, depending on the assay’s sensitivity. (This may include the influence of vaccination, as there is limited current evidence of the performance of antigen tests among vaccinated individuals.) Additionally, as variants of SARS-CoV-2 virus continue to emerge, the sensitivity of antigen tests for detecting prevalent variants may have a substantial impact on the performance of algorithms implementing antigen tests; however, early reports have found antigen tests perform similarly across multiple different SARS-CoV-2 variants [22]. However, only one report to date has evaluated the performance of immediate repeat antigen testing [13] and this may not be representative of settings where immediate repeat antigen testing performs with higher sensitivity. Importantly, this parameterization does not reflect the sensitivity of delayed repeat antigen testing (e.g. as recommended by ECDC for confirmation of negative results after 2-4 days when RT-PCR capacity is limited [8]). Finally, we adopted a simplifying assumption that NAATs have 100% sensitivity and specificity as NAATs are typically considered the “gold standard” for diagnosis of SARS-CoV-2 infection. However, NAATs may have lower sensitivity early in the course of infection [23] and remain positive during a patient’s post-infectious recovery [24]. Therefore, in our approach the prevalence among persons seeking testing is representative of currently and recently infected persons detectable by NAATs at the time of testing and some “missed cases” in this approach may represent post-infectious persons still detectable by NAAT.

## Conclusions

Our results provide the first quantitative comparison of the expected performance of different strategies for community-based SARS-CoV-2 testing programs recommended by public health organizations. None of the algorithms evaluated in this analysis is likely to be optimal in all settings and for all programmatic priorities, and this analysis provides a framework for selecting setting-specific strategies to achieve an acceptable balance and trade-offs between programmatic priorities and constraints. As global responses to the COVID-19 pandemic continue to evolve and adapt, our results contribute to the body of evidence informing SARS-CoV-2 testing strategies.

## Supplementary Information

**Additional file 1.**

## Data Availability

The dataset(s) supporting the conclusions of this article is (are) available in the CDC collaborative software GitHub repository (https://github.com/CDCgov/SARS-CoV-2-NAAT-and-Antigen-Testing-Algorithms).
